# *Saccharomyces boulardii* and *Bacillus subtilis* B10 modulate TLRs and cytokines expression patterns in jejunum and ileum of broilers

**DOI:** 10.1371/journal.pone.0173917

**Published:** 2017-03-20

**Authors:** Imran Rashid Rajput, Huang Ying, Sun Yajing, Muhammad Asif Arain, Li Weifen, Li Ping, Dost Muhammad Bloch, Liu Wenhua

**Affiliations:** 1 College of Science Shantou University, Shantou, Guangdong, P.R. China; 2 College of Animal Sciences, Zhejiang University, Hangzhou, Zhejiang, P.R. China; 3 Faculty of Veterinary and Animal Sciences, Lasbela University of Agriculture, Water and Marine Sciences, Uthal, Balochistan, Pakistan; 4 College of Animal Sciences and Technology, Northwest A&F University, Yangling, P.R. China; University of Calcutta, INDIA

## Abstract

The present study was designed to evaluate the effects of *Saccharomyces boulardii* (*Sb*) and *Bacillus subtilis* B10 (*Bs*) on intestinal epithelial Toll like receptors (TLR), and Cytokine expression response to understand the intestinal epithelial innate immune mechanism in broilers. A total of 300 birds (Sanhuang broilers) were allotted into three groups (n = 100) and each divided into five replications (n = 20). Control group (Ctr) birds were fed basal diet, broilers in experimental groups received (1×10^8^cfu/kg feed) *Sb* and *Bs* respectively in addition to basal diet for 72 days. The result showed significant increase in mRNA expression level of TLR2, TLR4 and TLR15. Down streaming MyD88, TRAF6, TAB2 and NF-κB mRNA level noted higher, in the jejunum and ileum as compared to control group. Meanwhile, IL-6, TNFα, IL-10, TGF-β expression levels showed high expression in the jejunum of *Sb* and *Bs* groups. IL-10 expression level increased in the ileum and IL-6, TNFα, IL-10 and TGF-β expression levels increased in the jejunum of *Sb* group. Levels of IL-1 β, IL-17, and IL-4, increased merely in *Sb* group. Ileal cytokines IL-1β, IL-17 and IL-4concentration were noted higher in *Sb* group, and IL-1β, and IL-4 levels were up-regulated in *Bs* group. The results indicated that the INF-γ and IL-8 level decreased in *Sb* and *BS* groups. Serum IgA and sIgA level increased in both treatment groups. Our findings illustrated that *S*. *boulardii* and *B*. *subtilis* B10 may have a role to induce mucosal immunity by activating the TLRs and cytokines expressions in broilers.

## Introduction

Intestinal surface is a complex and dynamic ecosystem that integrates an alliance among the epithelial barriers, immune mediators and myriads of microbes [1]. However, intestinal epithelium cells are not a physical barrier only, but also play an active role in the so-called ‘trilogue’ among luminal bacteria, epithelium and professional immune cells of lamina propria [2]. In addition, intestinal epithelium cells also discriminate the harmful and beneficial microorganisms [3]. They sense the microbes and/or their components through pattern recognition receptors (PRRs) and lead to the subsequent innate and adaptive immune responses. Toll-like receptors (TLRs) are a type of PRRs that can recognize a wide variety of microbial compounds and elicit immune activation [4]. Initially, innate receptors play an important role to balance the induction and reduction of inflammation in the host [5]. Simultaneously, it was reported that probiotics modulate the TLRs expression and induce cytokine’s production in the intestine [6], and this constant TLR stimulation may be necessary for maintaining intestinal health [7], although some degree of low level ‘surveillance’ NF-кB activation might be a normal physiological state [8].

Probiotics are defined as live microorganisms which, when administered in adequate amount exert beneficial effects on the host FAO/WHO [9], through improvements in the intestinal mucosa, where they interact and provide a primary barrier to defense against pathogens [10]. Moreover, probiotics have been shown to produce effects that stimulate multiple aspects of immune response, including activation of chicken TLRs in the gut [11], and modulation in cytokine production [12]. Another study also suggested that, TLRs show rapid adaptation of antigens exposure in a new environment and possible reflecting effects may appear, such as cytokines secretion with frequency-dependent response [13]. We reported that *S*. *boulardii* and *B*. *subtilis* B10 could modulate intestinal ultrastructure that might have influence to develop intestinal immunity [14]. But, the role of epithelium TLRs to induce intestinal immunity remained unknown. This evidence inspired us to focus our research on the role of probiotics (*Saccharomyces boulardii and Bacillus subtilis* B10) on mucosal immunity development through Toll like receptor activation. In continuance of previous research, we expended the study to know the effects of *Saccharomyces boulardii* and *Bacillus subtilis* B10 on the modulation of the epithelial TLRs expression and improvement in the mucosal immunity of broiler chickens.

## Materials and methods

### Ethics statement

This animal study (short title: Probiotics modulate intestinal immunity) was carried out in strict accordance with the recommendation of the National Ethical Commission (Zhejiang P.R. China). All procedures and experiments compile with the guideline and were approved by the local ethic committee of the Zhejiang University (Zhejiang Province, P.R. China) with respect to animal experimentation and care of animals under study, and all efforts were made to minimize suffering.

### Culturing of yeast and bacteria

Two different probiotics *Saccharomyces boulardii* (Yeast) and *Bacillus subtilis* B10 (Bacteria) were obtained from the Institute of Animal Nutrition and Feed Sciences, Provincial Key Laboratory of Feed Science, Zhejiang University selected for the trial to illustrate their effects. *S*. *boulardii* was cultured in Yeast Peptone Dextrose (YPD) broth (Oxoide; England) and *B*. *subtilis* in Luria Bertani (LB) broth (Oxoid; England). Separated probiotics were added in to the basal diet (Table 1), and concentration (1×10^8^cfu/Kg) was maintained accordingly [14]. In brief, *S*. *boulardii* and *B*. *subtilis* were separated by centrifugation at 6,000 × rpm for 5 min at 4°C and washed twice with PBS (pH 7.2–7.4). The latter concentration was determined using the optical density method (spectrophotometer, Lambda 850, Waltham, MA), and suspended in skim milk powder to prepare the required concentrations (1× 10^8^cfu/g), respectively. The prepared mixture was added into the basal diet (Table 1) and maintained at 1 × 10^8^cfu/kg.

**Table 1:** Composition and nutrition of the basal experimental diet (%).

Composition	1-36d	37-72d
Corn	55.90	61.60
Soybean meal	31.00	27.00
Wheat shorts	3.00	4.00
Imported fish meal	5.00	2.00
Rapeseed oil	1.50	2.00
Salt	0.30	0.30
Dicalcium phosphate	1.20	1.00
Limestone	1.00	1.00
DL-Met	0.10	
Lysine		0.10
Premix	1.00	1.00
Total	100.00	100.00
**Nutrient**		
ME (MJ/kg)	12.78	13.05
Crude protein	22.86	19.14
Lys	1.07	0.98
Met+Cys	0.86	0.72
Ash	7.38	6.41
Ca	0.93	0.91
Total phosphorus	0.64	0.56

Premix compound: Each kilogram contained: Vitamin A, 7 000 IU; VitaminD3, 2 500 IU; Vitamin E, 30 mg; VitaminK3, 1 mg; Vitamin B1, 1.5 mg; Vitamin B2, 4 mg; Vitamin B6, 2 mg; Vitamin B12, 0.02 mg; niacin, 30 mg; folic acid, 0.55 mg; pantothenic acid,10 mg; biotin, 0.16 mg; choline chloride, 400 mg; Cu, 20 mg; Fe, 70 mg; Mn, 100 mg; Zn, 70 mg; I, 0.4mg, Se,0.5 mg, virginiamycin 20mg/kg.

### Experimental design

A total of 300 -day old Chinese cross breed (Sanhuang broilers) was purchased from Yuan Yuan Poultry Hatchery Co. Ltd. The chicks were randomly allotted into three individual groups, and each group consisted of five replications (*n* = 20). Control group (Ctr) was fed a basal diet (Table 1), whereas birds in the experimental groups were fed basal diet with *S*. *boulardii* (*Sb*) and *B*. *subtilis* (*BS*) (1 × 10^8^cfu/kg) and experiment lasted for 72 days.

### Sampling procedure

The experiment was performed according to the guidelines provide by the Zhejiang Animal Center (ZAC) at Institute of Medical Sciences, and all of standard methods were officially approved by the ministry of livestock, Zhejiang University, Hangzhou, P.R China. Ten broiler chickens from each replicate were randomly selected and killed by administering lethobarb (0.5 mL/bird) intravenous (i.v). The jejunum and ileum were collected and opened longitudinally with micro scalpel, then transferred into a separate sterilized tube containing 10 M Phosphate buffer saline (7.4 pH, 1:4) and applied ultrasonic treatment for 4 min in order to separate the GUT contents from the GIT tissue which were accomplished by centrifugation (5000 rpm, 25 min at 4°C). After centrifugation, supernatant was utilized to determine cytokines.

### RNA extraction and cDNA synthesis

RNAiso (TaKaRa, Japan) was used to extract RNA from jejunum and ileal mucosal layer and purified using RNeasy^®^ MinEluteTM (TaKaRa, Japan) according to the manufacturer’s instructions. Total amount of RNA was quantified by Nano Drop (Thermo Scientific). Moreover, RNA was reverse transcribed using Reverse Transcriptase (RTase) M-MLV (RNase H^−^) (TaKaRa). Briefly, 1μg of RNA was combined with 10× first strand buffer, 1.0μl of oligo (dT) primer and RNase-free water up to 10 μl, mixed well and incubated at 70°C for 10 min. Moreover 5×M-MLV Buffer (4μl), 1μ1 of dNTP mix (10mM), RNase Inhibitor 0.5μl (40U/μl), RTase M-MLV 1 μ1 (200 U/μl) and RNase-free water to total volume of 20μl. The mixture was incubated at 42°C for 1h and 30 min; the reaction was stopped by heating at 70°C for 15min, and finally 5min on ice.

### Quantitative Real Time Polymerase Chain Reaction (qRT-PCR)

Oligonucleotide primers for chicken surface TLRs and β-actin were designed to evaluate the expressions, based upon sequences available from public databases (Table 2). ABI 7500 Real-time Detection System (Applied Biosystems, USA) was used to determine gene expressions at mRNA. The amplification was performed, containing 10ml of 2× SYBR Green I real-time PCR Master Mix (ABI), 1μl of the diluted cDNA, and 1.6 μl of mixed primer in a total volume of 20μl adding ddH2O. Denaturing was performed at 95°C for 15s, 60°C for 60s, followed by 40 cycles of 95°C for 15s and 60°C for 30s and dissociation step at 95°C for 10s, 60°C for 20 s, and 95°C for 15s. Moreover, comparative CT method was used, to determine gene expression, calculated as 2 ^ΔΔ^Ct [15].

**Table 2:** Real-time quantitative Reverse Transcriptase Polymerase Chain Reaction (qRT-PCR) primers sequences were used for gene expression.

Gene name	Sequence (5'-3') F: Forward R: Reverse	Accession numbers	Bp
**β-Actin**	F: 5'GAGAAATTGTGCGTGACATCA3'	JN639846.1	107
	R: 5'CCTGAACCTCTCATTGCCA3'		
**TLR1**	F:5'GGCAGTGGACGCAGACAAA3'	AB109401.1	89
	R:5'GTAGGAAATGAAGGCGTGGAA3'		
**TLR2**	F:5'CTGAAGCCACAGACATTCCTAAC3'	NM001161650.1	209
	R:5'CTTGTACCCAACGACCACCA3'		
**TLR4**	F:5'GGCAAAAAATGGAATCACGA3'	N001030693.1	201
	R:5'CTGGAGGAAGGCAATCATCA3'		
**TLR15**	F:5'ATCCTTGTCGTTCTGGTGCTAA3'	JN112029.1	187
	R:5'TCAGTAGATGCTCCTTCGTCCA3'		
**MyD88**	F:5'GGATGGTGGTCGTCATTTCA3'	NM001030962.1	226
	R:5'GAGATTTTGCCAGTCTTGTCCA3'		
**TRAF6**	F:5'CACAGAGGAGACGCAGGGATA3'	XM001235884.1	74
	R:5'AACAGATCGGGCACTCGTATTT3'		
**TAB2**	F:5'GAGTTTGCCAAGCAGACATCG3'	NM001006240.1	226
	R:5'GCACAGAGACTGGGTAGACACG3'		
**NF**_**κ**_**B**	F:5'ACCCCTTCAATGTGCCAATG3'	D13721	274
	R:5'TCAGCCCAGAAACGAACCTC3'		
**IL-6**	F:5'GCTCGCCGGCTTCGA3'	AJ309540	254
	R:5'GGTAGGTCTGAAAGGCGAACAG3'		
**TNF-α**	F: 5'GACATCCTTCAGCATCTCTTCA3'	JN942589.1	238
	R: 5'AGGCGCTGTAATCGTTGTCT3'		
**IL-10**	F:5'CGGGAGCTGAGGGTGAA3'	AJ621614	272
	R:5'GTGAAGAAGCGGTGACAGC3'		
**TGF-β**	F:5'CGGGACGGATGAGAAGAAC3'	M31160	258
	R:5'CGGCCCACGTAGTAAATGAT3'		

### Determination of cytokines by ELISA

The ELISA tests of IL-1β, (Biosource International, California, USA), IL-12, IL-17, IL-4, INF-γ and IL-8 (Genorise scientific Inc, Paoli, USA), were performed as manufacturer's instructions. Meanwhile, IgA, IgG, IgM and sIgA (Komabiotech, Ltd., Seoul, Korea), of serum were also performed as manufacturer’s guidelines. Briefly, polyclonal goat anti-chicken IL-1β, IL-12, IL-17, IL-4, INF-γ, IL-8, and IgA antibodies were applied as capturing antibodies, and biotinylated polyclonal goat anti-chicken IL-1β, IL-12, IL-17, IL-4, INF-γ, IL-8, and IgA antibodies as detecting antibodies. Streptavidin-RP and tetramethylbenzidine substrate (TMBS) were used as color indicator and subsequently color reaction was stopped with 2M sulfuric acid. Absorbance of each well was measured at (450 nm) wavelength, right after incubation at (37°C for 10 min).

### Statistical analyses

Data was analyzed using SPSS 16.0 for Windows (SPSS Inc, Chicago, USA) and expressed as mean ± standard deviation (SD). The intergroup variation was assessed by (ANOVA) followed by Fischer’s least significant difference (LSD) test. Statistical significance of the results was calculated at (*P*<0.05).

## Results

### Surface Toll like receptor and down streaming expressions response

**Fig 1:**
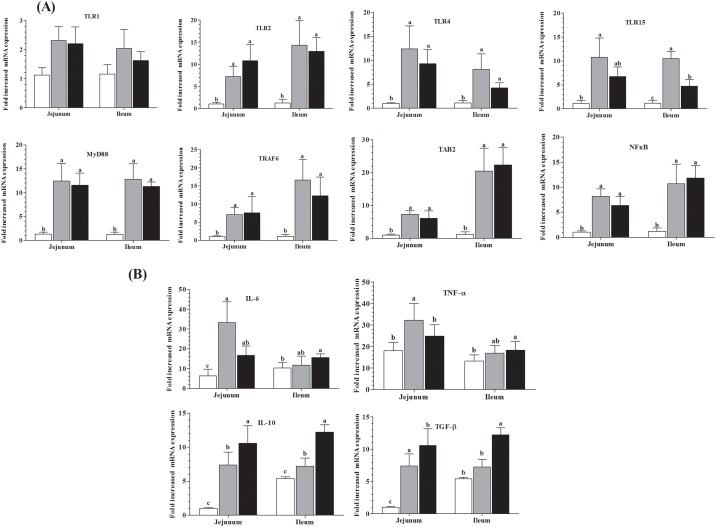
Effects of *S*. *boulardii and B*. *subtilis* B10 on jejunum and ileal mucosal epithelial cells mRNA expression levels. After oral administration of *Sb* and *Bs* (1x10^8^cfu/g), for seventy two days mRNA expressions of surface TLR1, TLR2, TLR4, and TLR15 (Fig. A) and downstream associated factors MyD88, TRAF6, TAB1 and NF_k_B (Fig. B) were evaluated. Data are presented as means of mRNA expression in fold change ± SD (n = 10). Means with different letters (a, b, c) are defined as significantly different (P < 0.05).

The present findings showed (Fig 1A) high mRNA expressions level of TLR2 (6.173 and 9.777) fold change increased in the jejunum and (13.054 and 11.643), fold change noted higher in the ileum of treatment groups. Moreover, mRNA expression level of TLR4 in the jejunum was (11.397 and 8.279) fold change higher, while in the ileum TLR4 expression (7.031) fold change increased in *Sb* group and (3.137) fold change in *Bs* group. TLR15 chicken specific receptor mRNA expression up-regulated in jejunum (9.608 and 5.594) and in the ileum (9.351 and 3.556) fold change in probiotic groups as compared with control group, respectively.

MyD88 is an adapter protein involved in the TLR signaling to develop innate immunity. In the present study, up-regulation of MyD88 mRNA expression (Fig 1) was observed (11.094 and 10.207) fold change in the jejunum and (11.543 and 10.006) fold change in the ileum of treatment groups, whereas TRAF6 expression level increased (P<0.05) in the jejunum 6.090 and 6.516-fold change and in the ileum 15.545 and11.132-fold change in both probiotic groups respectively. Moreover, treatment groups improved TAB2 gene expressions in jejunum (6.2976 and 5.078), and in the ileum (19.304 and 21.124) fold change. Transcription complex NF-κB mRNA levels showed high intensity of expression in jejunum (7.151 and 5.303) and (9.500 and 10.689) fold change in the ileum of probiotic groups as compared with Ctr group.

### Cytokines expressions response

Cytokines productions are known as a response of immune cells. The results of treatment group showed (Fig 1B), that IL-6, TNFα, IL-10 TGF-β, expression levels significantly up-regulated in the jejunum of *Sb* and *Bs* groups. Meanwhile, IL-10 was noted higher in the ileum of *Sb* group and *Bs* group had increased expression level of IL-6, TNFα, IL-10 and TGF-β in the ileum as compared to control.

### Effect of probiotics on cytokine production in jejunum and ileum

**Fig 2:**
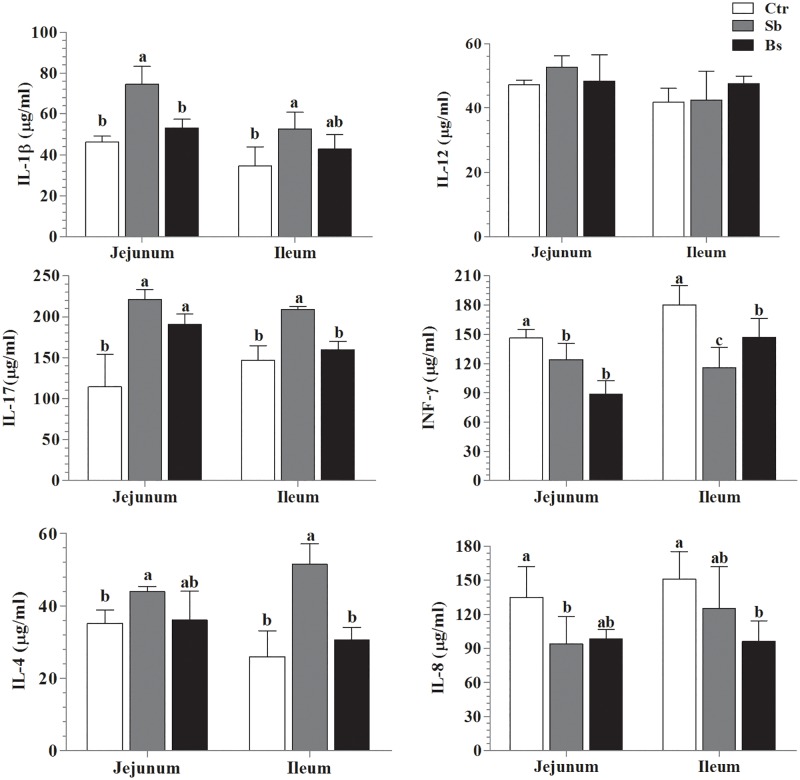
Effects of orally administered probiotics (1x10^8^cfu/g), *Saccharomyces boulardii* (*Sb*) and *Bacillus subtilis* (*Bs*), on jejunum and ileal cytokines IL-1β, TNF-α, IL-12, IL-17, IL-6, IL-4, IL-10 TGF-β, INF-γ and IL-8 secretion level was determined by ELISA, and fixed optical density (450nm) was applied. The results are represented as Means± SD (n = 10). Different letters (a, b, c) show significantly difference (P < 0.05), among groups.

We quantified the cytokine levels to evaluate the effects of probiotics (*S*. *boulardii*and *B*. *subtilis*) in the jejunum and ileum of broiler chickens (Fig 2), *S*. *boulardii* treatment group showed marked increase in the secretion of IL-1β, IL-17 and IL-4, whereas *Bs* group significantly improved IL-17, in the jejunum. Conversely, IL-8 and INF-γ levels were down-regulated in the jejunum of both treatment groups. However, IL-1β, IL-17, and IL-4 concentration increased in the ileum of *Sb* group, while IL-4 concentrations were noted higher in *Bs* group. INF-γ was down-regulated in both (*Sb* and *Bs*) groups and IL-8 concentration level dropped in *Bs* group only as compared with Ctr group in the ileum.

### Effects of probiotics on IgA and sIgA concentration in jejunum and ileum

**Fig 3:**
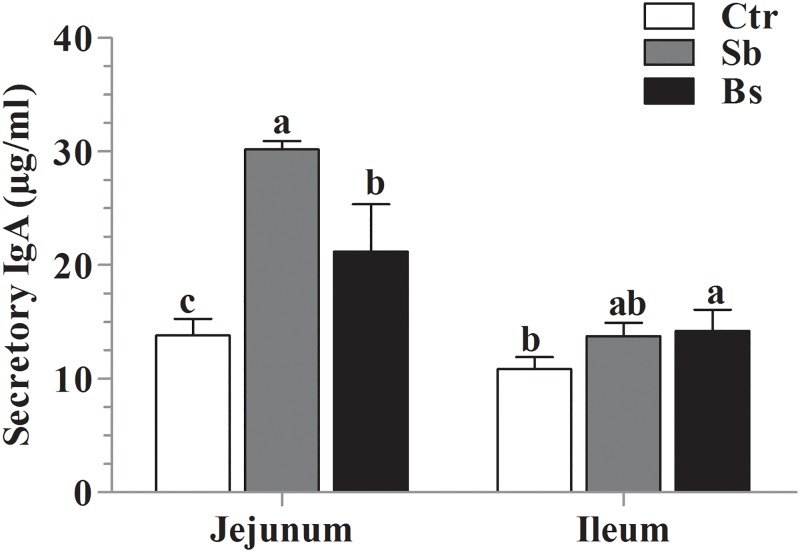
Effects of *Saccharomyces boulardii* and *Bacillus subtilis* on jejunum and ileum sIgA secretion levels. The results presented mean ± SD, of (n = 10) broiler chickens and different letters, a, b and c show significant difference (P < 0.05) among the groups.

The results showed that (Fig 3) sIgA concentration improved in the jejunum of *Sb* and *Bs* groups, but in the ileum *Bs* group increased sIgA only, and *Sb* group remained unchanged in comparison of Ctr group.

### Serum immunoglobulin assay

**Fig 4:**
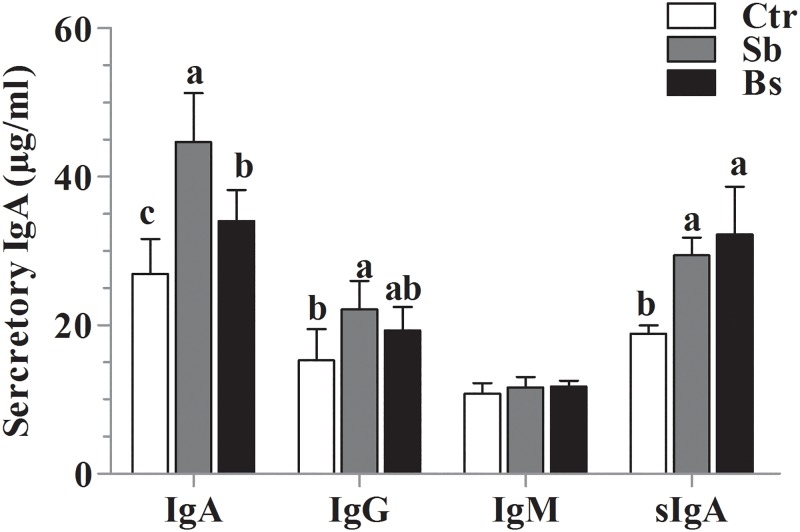
Effects of *S*. *boulardii* and *B*. *subtilis* on serum cytokines secretion level of Immunoglobulin (IgA), Immunoglobulin G (IgG), Immunoglobulin M (IgM) and secretary Immunoglobulin A (sIgA). Values are mean ± SD, different letters, a, b and c show significant difference (P < 0.05) among the groups (n = 10).

The concentration of immunoglobulin in serum showed (Fig 4) that serum IgA and sIgA increased (*P*<0.05) in *Sb* and *Bs* treatment groups. However, IgG was induced in *Sb* group only, and IgM did not show any increase as compared to Ctr group.

## Discussion

Probiotics provide opportunities to apply the beneficial microbes to human or animals (or both) for initial health and clinical applications. Therefore, it is essential to understand the underlying mechanism of probiotics to develop mucosal immunity via intestinal TLRs and cytokines expression in broilers. TLRs are initial signaling tool to activate immune responses to regulate intestinal cells, specifically epithelial cells proliferation protecting against harmful microbes and maintaining the homeostasis of the host [7]. In the present study, after oral administration of *S*. *boulardii* and *B*. *subtilis* B10, mRNA expression level of TLR2, TLR4 and TLR15 increased in the jejunum and ileum of broiler chickens. Similarly, administration of *C*. *albicans* and *S*. *boulardii* up-regulated TLR2 and TLR4 gene expressions in the colon, moreover chicken TLR2 (chTLR2) expression level increased after stimulation with probiotics [4, 16]. Microbiota isolated from intestine also up regulated the expression levels of TLR2, TLR3, TLR4 and TLR5 [17]. In another study, application of synthesized MALP-2 (a diacylated lipoprotein from Mycoplasma) to HEK-293 cells improved the expressions of chTLR2 and resulted in NF-κB activation [18]. Chicken specific surface receptor TLR15 expression was up regulated in chicken fibroblasts after incubation with heat-killed bacteria [19]. In addition, heat killed heterophils gram-negative or gram-positive bacteria increased chTLR15 expressions [20]. Administration of probiotics stimulated TLRs and modulated cytokine production to activate cellular innate immunity [21]. Recently, it was found that TLR15 does not bind a ligand, but instead is stimulated following its proteolytic cleavage by virulence-associated proteases [22]. This presents itself as novel mechanism of TLRs activation unique to the chickens, and future studies should be aimed at characterizing the immunomodulatory properties of these virulence-associated proteases [23]. Triggering of TLRs by probiotics initiates an innate immune response that activates pro-inflammatory mediators and afterward leads to the recruitment of inflammatory cells and initiates an appropriate response to develop innate immunity [24]. In addition, TLRs activation also has an important role to enhance the trans-epithelial resistance against invading bacteria [25]. Our findings suggest that chicken TLRs and avian specific TLR15 stimulation through *S*. *boulardii and B*. *subtilis* might play a key role to induce innate immunity in the intestine of broiler chickens.

MyD88 is an adapter protein and has an effective role in downstream signaling pathway to activate the innate immune response via TRAF 6 and TAB1 mediated proteins that lead to the activation of NF-κB [26]. In our study, application of probiotics markedly increased the expression levels of MyD88, TRAF6, TAB1 and NF-κB in the jejunum and ileum, whereas ileum was found more responding than jejunum. Similarly, administration of *B*. *breve* developed the immunity depending on MyD88 pathway, and it showed suppression of intestinal inflammation via induction of IL-10-production [23]. Previous studies indicated that, MyD88, TLR2, or TLR4 deficient mice were highly sensitive to intestinal inflammation [7, 27]. However, probiotics trigger MyD88-dependent signaling pathway to activate the immune system in the gut Takeuchi et al. [28] by stimulating TLRs and subsequently TRAF6. It is an important associated protein consisting of TRAF-N terminal and TRAF-C terminal that plays a crucial role to mediate the cytokine signals [29]. It dissociates from the receptor and associates with TAB1 and TAB2, where the complex of TRAF6, TAB1 and TAB2 moves into the cytoplasm and make large complex with other proteins [30]. Thereafter, a lysine 63-linked polyubiquitin of TRAF6 is synthesized that induces TRAF6 mediated triggering of TAB1/2 and finally activation of NF-κB Deng et al. [31] and similar mechanism of response we observed in the present study.

Inflammatory cytokines endorse cell mediated immunity allied to Th1 cells [32], and cytokines secretion level depends on probiotic species [33]. In our study, *Sb* increased the production of IL-1β, IL-17 and IL-4, whereas *Bs* group significantly improved IL-17, in the jejunum. IL-1β, IL-17 and IL-4 showed higher levels in the ileum of *Sb* and *Bs* groups. Meanwhile, concentration of IL-1β, and IL-4, were noted higher in *Sb* and *Bs* groups respectively; however, IL-8 and INF-γ were down-regulated in both groups. In another study, it was found that application of *B*.*breve* improved IL-10 concentration in the colonic tissues of SCID mice [34], whereas IL-8 and IFN-γ concentration was down-regulated in Caco-2 cells [35]. Moreover, *Clostridium* species have been shown TGF-β induction in the colon through the epithelial cells [36]. Inflammation is characterized by the initial release of pro-inflammatory cytokines (IL-1 and INF-γ) and chemokines [37], afterword IFN-γ, IL-12 and IL-17 are involved in host defense against infection. Similarly, regulation of pro-inflammatory cytokines is controlled by the release of IL-10, therefore ups and downs inTh1 inflammatory responses [38], due to IL-4, and TGF-β initially up and later down-regulates during infection [39]. Our findings clearly demonstrate that probiotic strains (*S*. *boulardii* and *B*. *subtilis* B10) promote the epithelial cell cytokines level to develop mucosal immunity.

Systemic response of antibodies in the serum via soluble antigens could be induced by probiotics and that might play crucial role to increase the innate immunity [40]. The present study showed that serum IgA and sIgA increased in *Sb* and *Bs* treatment groups. However, IgG induced in *Sb* group only. Similarly, administration of probiotics containing *Lactobacillus acidophilus* and *Lactobacillus casei* enhanced the serum IgA and IgG response [41]. Application of probiotics stimulates immune cells and subsequently cytokine production [42]. It was interesting that *S*. *boulardii* and *B*. *subtilis* probiotics increased the level of immunoglobulins in serum of broiler chickens.

The IgA isotype is primary protective immunoglobulin of intestinal secretions and is synthesized in lamina propria by plasma cells to defend and regulate the homeostasis [43]. Similarly, an enhanced IgA and IgG concentration was reported after administration of probiotics containing *Lactobacillus acidophilus* and *Lactobacillus casei* [42]. Intestinal commensal bacteria are major participant in the synthesis of mucosal IgA, because IgA is absent in the intestine of germ free animals [44]. However, a fraction of IgA antibodies is also generated in T-cells by independent manner [45]. Moreover, IL-6, IL-10 and TGF-α promote class switching of B cells to produce IgA [46], that activate and proliferate by interactions with local environmental T cells; additionally, unknown structural components of gram-positive bacteria are also responsible for elicitation of intestinal IgA antibodies [43]. In the present study similar results were found that *S*. *boulardii* and *B*. *subtilis* B10 could improve the sIgA level in the intestine of broiler chickens.

## Conclusion

In conclusion, oral administration of probiotics strains *Saccharomyces boulardii* and *Bacillus subtilis* B10could modulate the mRNA expression of intestinal TLRs and down streaming associated factors, while improve production of cytokines to endorse the mucosal immunity.

## Abbreviations

*Bs*: *Bacillus subtilis* B10
Ctr: Control group
IgA: Immunoglobulin
IL-1β: Interlukin-1beta
INF-γ: Interferon gamma
LB: Luria Bertani
MyD88: Myeloid differentiation 88
NF-κB: Nuclear factor kappa-B
*Sb*: *Saccharomyces boulardii*
sIgA: Secretory immunoglobulin
TAB2: TGF-beta activated kinase binding protein2
TGF-β: Transforming growth factor beta
TLR: Toll like receptors
TMBS: Tetramethylbenzidine substrate
TNFα: Tumor necrosis factor alpha
TRAF6: TNF receptor associated factor-6
YPD: Yeast Peptone Dextrose
