# Correction: *Saccharomyces Boulardii* and *Bacillus Subtilis* B10 modulate TLRs and cytokines expression patterns in jejunum and ileum of broilers

**DOI:** 10.1371/journal.pone.0180752

**Published:** 2017-06-29

**Authors:** Imran Rashid Rajput, Huang Ying, Sun Yajing, Muhammad Asif Arain, Li Weifen, Li Ping, Dost Muhammad Bloch, Liu Wenhua

The affiliation for the last author is incorrect. Liu Wenhua is not affiliated with #2 but with #1 College of Science Shantou University, Shantou, Guangdong, P.R. China.

There are errors in the caption for Fig 1. Please see the complete, correct Fig 1 caption here.

**Fig 1:**
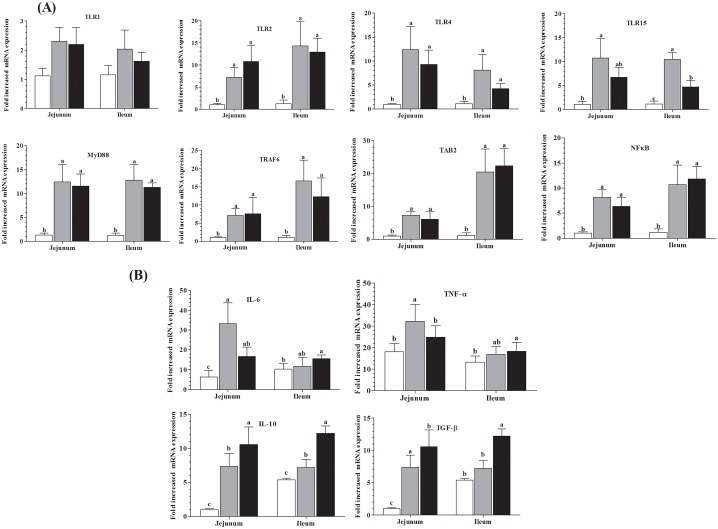
Effects of Sb and Bs B10 on jejunum and ileal mucosal epithelial cells mRNA expression levels. Fig A. After oral administration of Sb and Bs (1x108cfu/g), for seventy-two days mRNA expressions of surface TLR1, TLR2, TLR4, and TLR15 and downstream associated factors MyD88, TRAF6, TAB2 and NFkB were evaluated. Fig B. Jejunum and ileal cytokines mRNA expressions of IL-6, TNF-α, IL-10 and TGF-β were determined by qRT-PCR. Data are presented as means of mRNA expression in fold change ± SD (n = 10). Means with different letters (a, b, c) are defined as significantly different (P < 0.05).
